# Permissive azotemia during acute kidney injury enables more rapid renal recovery and less renal fibrosis: a hypothesis and clinical development plan

**DOI:** 10.1186/s13054-022-03988-0

**Published:** 2022-04-28

**Authors:** Lakhmir S. Chawla

**Affiliations:** Department of Medicine, Veterans Affairs Medical Center, 3550 La Jolla Village Drive, San Diego, CA USA

## Abstract

Preclinical models of acute kidney injury (AKI) consistently demonstrate that a uremic milieu enhances renal recovery and decreases kidney fibrosis. Similarly, significant decreases in monocyte/macrophage infiltration, complement levels, and other markers of inflammation in the injured kidney are observed across multiple studies and species. In essence, decreased renal clearance has the surprising and counterintuitive effect of being an effective treatment for AKI. In this Perspective, the author suggests a hypothesis describing why the uremic milieu is kidney protective and proposes a clinical trial of ‘permissive azotemia’ to improve renal recovery and long-term renal outcomes in critically ill patients with severe AKI.

## Introduction

Preclinical models of acute kidney injury (AKI) consistently demonstrate that a post-kidney injury background of adequate renal clearance exacerbates fibrosis and delays renal recovery of the injured kidney [1–8]. Conversely, contralateral nephrectomy prior to unilateral kidney injury decreases fibrosis and enhances renal recovery in the injured kidney [8, 9]. Similarly, delayed contralateral nephrectomy of the healthy kidney after unilateral kidney injury decreases fibrosis and accelerates recovery of the injured kidney [8, 9]. Notably, the effect of decreased renal clearance induced by nephrectomy of the healthy kidney initiates a profound change in the contralateral injured kidney whether the injury is due to ischemia/reperfusion (IR) or unilateral ureteral obstruction (UUO) [8, 10]. Significant decreases in monocyte/macrophage infiltration, complement levels, oxidative stress, and other markers of inflammation in the injured kidney are observed in addition to improved histology and less fibrosis [8, 10, 11]. These findings have been consistently demonstrated across multiple animal species in the multiple laboratories across the globe. In essence, decreased renal clearance leading to a uremic milieu has the surprising and counterintuitive effect of being an effective treatment for severe AKI.

## Previous work

**Fig. 1:**
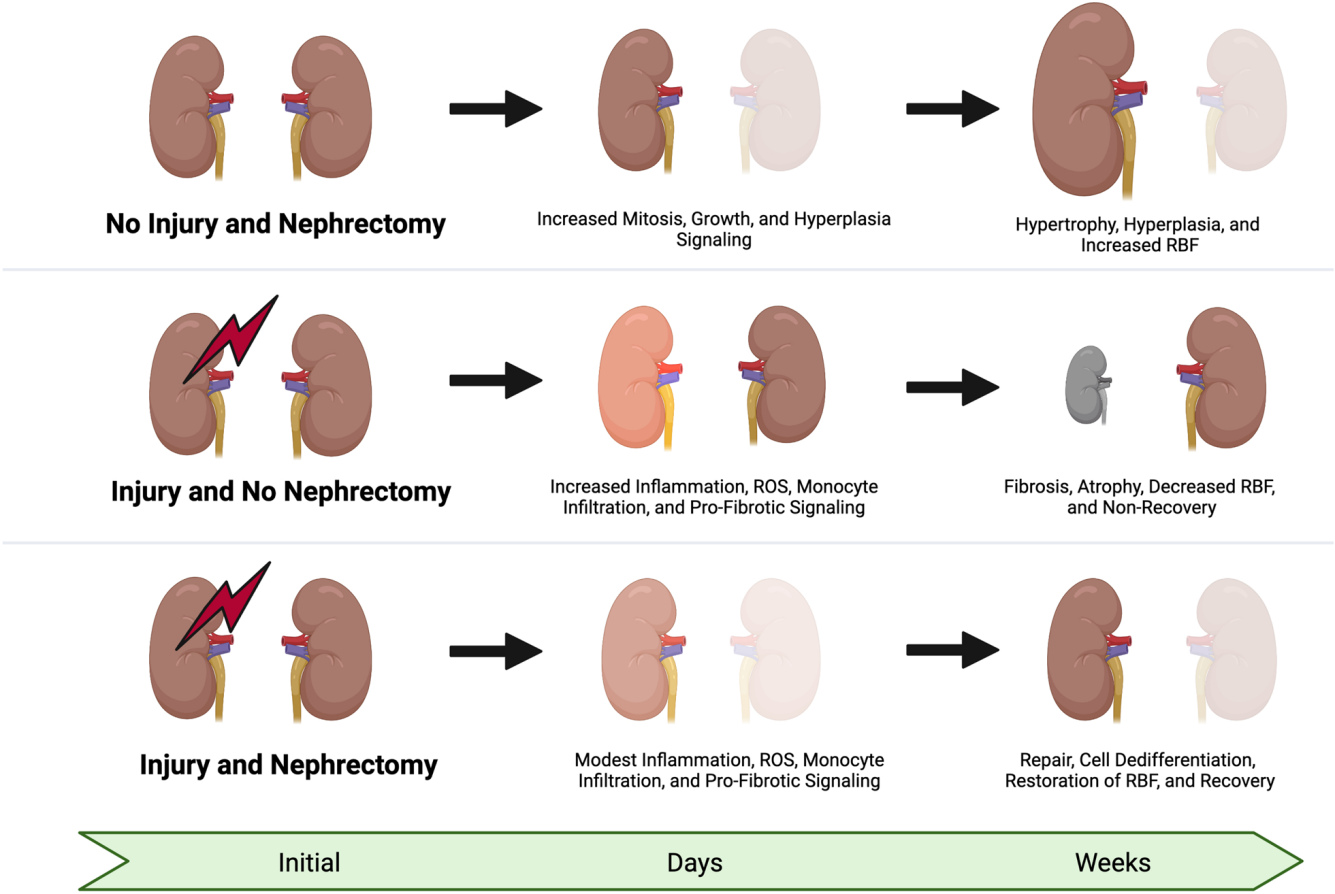
Effect of nephrectomy on kidney recovery after acute kidney injury

In 1954, Koletsky and then Finn in 1980 reported that unilateral renal IR injury led to progressive functional impairment and fibrosis of the kidney over a 2-week period [1, 2]. If the contralateral healthy kidney was removed, the injured kidney demonstrated functional recovery. In the decades since, the protective effect of nephrectomy of the uninjured contralateral kidney is robust and reproducible. As compared to non-nephrectomized animals, contralateral nephrectomy in unilateral injured animals showed improved histology as evidenced by less severe necrosis, fewer casts, and less apoptosis [3, 4, 7]. In addition to improved histology, the inflammatory response is lessened by nephrectomy along with lower levels of oxidative stress [7, 8, 11]. The decreases in inflammation and oxidative stress are mirrored by a blunting of the pro-fibrotic response with a marked downregulation of the fibrotic cellular genetic response and decreased activation of pericytes and myofibroblasts [7, 8, 11]. Also, the renal blood flow to the injured kidney recovers more rapidly and is enhanced by nephrectomy [7, 12, 13]. These aggregate effects from the uremic milieu are initiated by nephrectomy and lead to both structural and functional renal recovery in the injured kidney. Whereas failure to conduct a nephrectomy leads to atrophy and fibrosis in the injured kidney, nephrectomy prior to injury leads to the earliest recovery [8, 13]. Delayed nephrectomy after injury still leads to recovery, which is possible as far out as 2 weeks [8] (Fig. 1).

Based on these findings, one possible explanation is that the surgical procedure of nephrectomy induces the protective effect through preconditioning. However, through a large body of previous work, it has been well established that the uremic milieu is responsible for these findings. Firstly, delayed nephrectomy after initial contralateral IRI results in enhanced recovery. Second, the protective effects on the injured kidney are also seen when the uninjured kidney is rendered dysfunctional non-surgically, such as through ureteral transection [3, 14]. Third, these findings can also be demonstrated in vitro wherein peritoneal dialysate from uremic mice was added to cell culture, leading to the development of cyto-resistance to injury of proximal tubular cells [14]. Lastly, and perhaps most directly, urea loading done prior to renal artery occlusion conferred significant renal protection [15].

Zager et al. conducted an elegant series of experiments that clearly showed that the uremic milieu was responsible for kidney protection [6, 14, 15]. Further, the uremic milieu effect on renal recovery was dose dependent. The more severe the uremic milieu, the better the renal recovery. In one study, Zager et al. created a graded uremic milieu background and then assessed the impact on a unilateral kidney subjected to 30 min of IR injury. In this study, they demonstrate, in a stepwise fashion, that the higher the blood urea nitrogen (BUN)—the better the recovery of the injured kidney [6]. The findings of this study and many other similar studies are consistently reproducible and have led some investigators to wonder whether there is a ‘drug’ hiding in the uremic milieu. Perhaps this uremic factor could be isolated and purified leading to a drug for the treatment of AKI?

The notion of finding a uremic factor to develop as a drug is an attractive concept. Substantial research effort has been done, and the field continues to catalog uremic toxins. The European Uremic Toxin Work Group (EUTox) has created a process by which uremic toxins are classified by small water-soluble toxins, protein-bound toxins, and middle molecules [16]. In addition, each of the identified toxins is assessed for their toxic capacity and the link if any to clinical outcomes. Thus far, well over 100 such toxins have been identified and with advanced analytical techniques, more are being discovered [16]. The notion of finding the uremic toxin(s) that are responsible for the protective effects is appealing, but developing these molecule(s) as drugs could be difficult as each toxin would need to be produced at scale in order to test that molecule as a therapeutic agent.

Many toxins have been identified in the uremic milieu, with the most toxic identified thus far as 3 water-soluble compounds [asymmetric dimethylarginine (ADMA), trimethylamine N-oxide (MAO), uric acid], 6 protein-bound compounds [advanced glycation end products (AGEs); p-cresol sulfate; indoxyl sulfate; indole acetic acid; the kynurenines; phenyl acetic acid] and 3 middle molecules [*B*_2_-microglobulin; ghrelin; parathyroid hormone) [16]. Urea itself can be toxic and has been shown to induce disintegration of the gut epithelial barrier which can lead to translocation of bacterial toxins into the bloodstream [17]. Urea can induce apoptosis of vascular smooth muscle cells which can cause endothelial dysfunction, and urea can stimulate oxidative stress and dysfunction in adipocytes, leading to insulin resistance [17]. Also, there are indirect effects of elevated urea as a result of the carbamylation reaction, where isocyanic acid (a product of urea catabolism) alters the structure and function of proteins in the body [17].

It is well established that renal inflammation after kidney injury is associated with worse outcomes and more severe fibrosis; thus, an increase in uremic toxins would be assumed to be harmful in AKI. Because urea itself and the uremic milieu have been shown to be inflammatory and harmful, these findings of its protective properties on kidney injury seem contradictory [16, 18].

## The open and unresolved question: How does the uremic milieu confer kidney protection?

### Proposed hypothesis

**Fig. 2:**
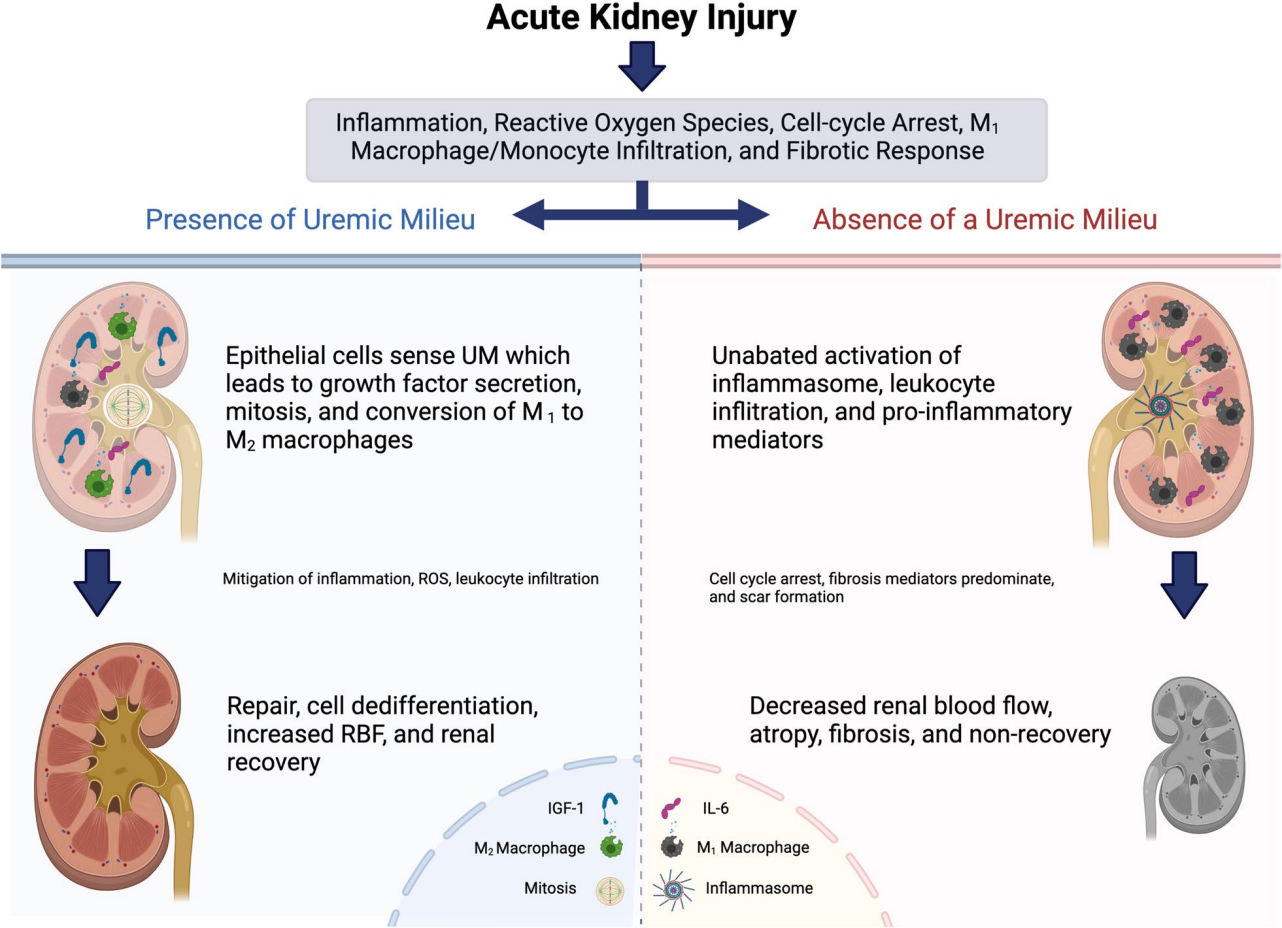
Proposed mechanism of action: regenerative effects of uremic milieu on renal recovery after acute kidney injury

While uremia itself is can be pro-inflammatory, the uremic milieu is a strong stimulus for renal cellular regeneration and repair. This pro-survival and growth signaling is initiated by renal cells that can sense a uremic milieu, thus increasing intracellular, autocrine, and paracrine signaling of pro-survival/growth factors in kidney cells. These effects in turn lead to mitosis and regeneration while simultaneously altering the renal microenvironment, thus decreasing leukocyte infiltration and inflammation, which therefore enhances repair and recovery (Fig. 2).

Thus, while the uremic milieu can be inflammatory, this effect takes time as the uremic toxins build up, whereas the immediate effect of increased uremic toxin levels is potent pro-survival signaling, thereby activating an improved renal microenvironment and enhanced renal blood flow, which, taken together, generates improved renal outcomes. Simply put, in the acute setting, the uremic milieu does more good than bad for the injured kidney.

### Evidence supporting the proposed hypothesis

It is well established in preclinical models and human studies that unilateral nephrectomy results in an increased uremic solute burden, which results in hypertrophy of the remaining healthy contralateral kidney [7]. Preclinical studies show that post-nephrectomy, the cellular response of the contralateral uninjured kidney is induction of cell cycle progression, hypertrophy, and mitosis [7]. Of note, this effect on the remaining kidney occurs within hours. Consistent with this response, the pro-survival growth factors such as EGF, HGF, and IGF-1 are seen in the remaining kidney [4, 19, 20]. In aggregate, the uremic milieu induces hyperplasia and hypertrophy in the remaining healthy kidney after contralateral nephrectomy which is a natural and adaptive response to the loss of the renal function. In vitro work shows that proximal tubular cells develop cyto-resistance when exposed to a uremic milieu, further supporting the argument for direct cellular sensing and response to uremic toxins [14].

While the precise mechanism by which renal cells sense the uremic milieu has not been identified, one obvious answer is urea itself. In animal models, urea loading prior to renal ischemia has been shown to be protective [15]. The protective effect urea loading effect was similar whether combination of urea and creatinine or human urine was used [15]. In addition, it is well documented that renal tissue possesses multiple urea transporters (UT) and these transporters allow renal cells to sense the urea concentration [21]. In particular, UT-A is predominantly expressed by renal epithelial cells. The author speculates that as the urea concentration increases due to kidney injury, the renal epithelial intracellular urea concentration also increases which likely signals the adaptive response [21]. If urea alone is found to be the primary driver of these protective effects on an injured kidney, urea could be delivered as drug by oral or intravenous dosing.

As outlined in Fig. 2, the author proposes that the uremic milieu is a strong inducer of renal survival and growth. These cellular responses are from direct sensing of uremic milieu leading to the production of various growth factors, cell proliferation, and survival signaling which are imparted in the renal microenvironment [14, 22, 23]. These paracrine effects lower the levels of inflammation, oxidative stress, and the degree of monocyte/macrophage infiltration while converting macrophages and monocytes from their M_1_ phenotype to an M_2_ phenotype. By stimulating a growth, mitosis, and repair response, renal blood flow improves more rapidly, thus facilitating a virtuous cycle of repair and recovery leading to more rapid return of renal function.

The mammalian evolutionary adaptive response to stress is well preserved, and there are many examples of ‘harm’ signals that induce function. Hypotension is potent stimulus of ADH and renin release, and hypoglycemia is a potent stimulus for sympathetic tone and glucocorticoid release. Just as in pulmonary physiology, a rising CO_2_ level is a potent stimulus to breath; similarly, the uremic milieu is potent stimulus for the kidney to filter.

Clinical trials of RRT timing (early vs delayed vs more delayed) do not offer any clear indication of whether permissive azotemia is potentially beneficial [24–26]. In AKIKI-2, no clear benefit was seen with delayed RRT, but there was a trend toward fewer RRT-free days [24]. However, AKIKI-2 did show that a more delayed strategy may cause harm. RRT timing studies are not ideal for this assessment, and dose comparison studies are required. A study done in Japan by Fujii and colleagues showed that a reduced CRRT dose (below median) had a trend toward improved survival compared to a higher-dose CRRT (median dose 16 ml/kg/h) [27]. While not definitive, these data suggest that a lower clearance approach can be undertaken safely.

### Testing the hypothesis

Patients with severe AKI requiring RRT may benefit from an *initial* RRT prescription which fosters permissive azotemia. Specifically, the prescribed clearance is minimal and targets a BUN of 100–150 mg/dL. Importantly, although the prescribed clearance is low, the RRT prescription still allows for prompt and active treatment of fluid overload (FO), electrolyte disorders, and acid/base imbalances. In general, the proximate harm caused by severe AKI is due to fluid overload, electrolyte disorders, and/or acidemia; these metabolic and volume defects can be treated with RRT while still achieving a minimal clearance.

The author recognizes that suggesting an RRT prescription that deliberately permits azotemia is heresy and lies in stark contrast to the consensus guideline and collective dogma of RRT treatment in severe AKI [28]. However, there is an important dichotomy of treatment paradigm that differs for acute versus chronic disease. For example, chronic kidney disease benefits from angiotensin II blockade, but for patients with AKI due to vasodilatory shock, the addition of angiotensin II has been shown to have benefit in patients with AKI [29]. Similarly, congestive heart failure outcomes are worsened by inotropes such as milrinone and catecholamines, but these inotropes are lifesaving for patients with acute cardiac dysfunction after cardiac surgery [30]. Thus, during acute illness, the treatments that are established for chronic disease are often mechanistically opposite to the appropriate therapy in acute illness.

## Clinical development and a study protocol

The initial step to test this hypothesis would be to conduct a pilot trial that shows an *initial* RRT prescription that targets an elevated BUN (e.g., BUN 100–150 mg/dL) can be performed safely while still appropriately treating FO, electrolyte disorders, and acid/base imbalances. A study comparing a dose 10–15 ml/kg/min compared to 20–25 ml/kg/min would be a reasonable first pilot trial. If this is proved non-inferior, additional lowering of the clearance could be contemplated. In order to maintain a high BUN, an RRT prescription of 3–5 ml/kg/h (vs. the conventional 20–25 ml/kg/h) while still treating FO, acidemia, and electrolyte disorders could be undertaken. Patients with severe acidemia, catabolic disorders, concomitant ingestions, or severe hyperkalemia should be excluded from such a trial. Some patients may require an initial period of enhanced clearance to control acidemia and electrolyte disorders.

Once a safe clinical prescription for permissive azotemia is established by pilot study(ies), larger clinical trials can be conducted. The expected benefits that should be seen based on this hypothesis are manifold. Based on the preclinical data, 7–10 days of permissive azotemia should result in more rapid renal recovery, less kidney fibrosis, and less inflammation. Importantly, as a matter of study conduct, the uremic milieu should not be imposed beyond 7–10 days. Similarly, if the uremic milieu has been established for 7–10 days and renal recovery has not occurred, increased RRT clearance should be reestablished gradually in order to avoid disequilibrium effects. In addition, since the largest costs of RRT are the duration of RRT and the cost of dialysate fluids, if this approach was shown to be beneficial, it would also be associated with lower costs as related to fewer days of RRT and less fluid required.

It is important to consider that while the author advocates a state of permissive azotemia, the author does not advocate an approach of delayed initiation of RRT. For many, severe hyperkalemia and severe acidemia are proximate causes of the harm due to severe AKI. However, these indications for the initiation of RRT are less common. In the view of the author, FO is the more insidious harm from severe AKI and is often treated late. For purposes of this clinical study plan, FO causing pulmonary edema and/or venous congestion should be treated promptly. As such, since the BUN will be allowed to rise with this approach, the clinical protocol should focus on FO, electrolyte disturbance, and/or acidemia for the decision to initiate RRT.

## Summary

Preclinical data consistently demonstrate that the uremic milieu improves renal recovery in AKI and acts as a strong inducer of renal repair and recovery. While the sustained uremic milieu itself can be inflammatory, the author proposes a clinical development protocol wherein an initial and limited trial of permissive azotemia is prescribed for patients with severe AKI requiring RRT. The goal of this approach is to harness the uremic milieu to augment renal recovery while treating FO, electrolyte disorders, and acid/base imbalances. The anticipated benefits of this approach would be more rapid renal recovery, decreased renal fibrosis, and decreased costs associated with RRT.

## Data Availability

All information in the Perspective is based on published literature.
